# Cutaneous Ciliated Cyst in an Unusual Location: Between Two Scapulas

**DOI:** 10.1155/2018/5961913

**Published:** 2018-04-01

**Authors:** Gül Doğan, Hülya İpek, Mehmet Metin, Özgür Özkayar, Çağatay E. Afşarlar

**Affiliations:** ^1^Department of Pediatric Surgery, Hitit University Erol Olcok Training and Research Hospital, Çorum, Turkey; ^2^Department of Pediatric Surgery, Hitit University Faculty of Medicine, Çorum, Turkey

## Abstract

Cutaneous ciliated cyst is defined as a rare, painless lesion frequently encountered on the lower extremities of young girls after puberty. The cyst is surrounded by the columnar ciliary epithelium. Apart from the lower extremities of girls, they may be localized on the scalp, scapula, thumb, abdomen, umbilicus, thigh, heel, knee, and gluteal region. There are two theories to explain this localization. The first is that they are mullerian heterotrophy, while the other is that they are ciliated metaplasia of eccrine glands. In this paper, we described a cutaneous ciliated cyst, which was observed with a previously undescribed localization on the back of a 13-year-old female patient.

## 1. Introduction

Cutaneous ciliated cyst is an exceptional lesion. It is more frequent in girls after puberty and usually manifests with a painless mass located on lower extremities [1, 2]. The scalp, scapula, thumb, abdomen, umbilicus, perineum, inguinal region, thigh, ankle, knee, and gluteus are other locations of cutaneous ciliated cysts reported in girls [1, 3]. Additionally, cheek, scrotum, and shoulder locations were reported in boys [4]. We reported a case of cutaneous ciliated cyst located on the back between two scapulas in a 13-year-old girl.

## 2. Case

**Figure 1:**
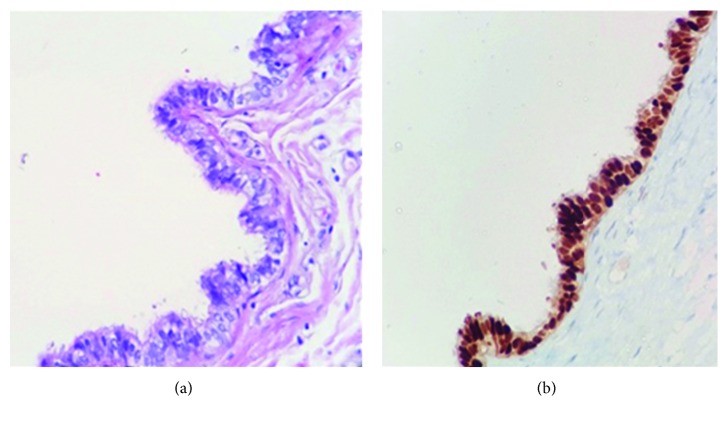
(a) Pseudostratified cilia columnar epithelium resembling tubal epithelium along with occasional small projections toward the lumen in epithelium of the cyst wall (H&E stained section, 400x). (b) Immunohistochemical study demonstrates that the cyst epithelium is nuclear positive for PR and negative for CEA, and SMA staining is negative within the cyst wall indicating no smooth muscle in the cyst wall (400x).

A 13-year-old female patient was brought to the pediatric surgery clinic due to a swelling on the midline of the back that had not resolved for a few months. Physical examination revealed a soft-textured, fluctuating, nonhyperemic, painless mass roughly 2 × 1 cm in size on the midline of the back between the two scapulas. Surface ultrasonography demonstrated a 20 × 7 mm regular-contoured cystic lesion containing septations under the skin. After obtaining parental and patient's informed consent for the treatment and agreement for participating in medical research, surgical excision was justified. The mass was totally excised, and the postoperative course was uneventful. On histopathologic investigation of the specimen, pseudostratified cilia columnar epithelium resembling normal tubal epithelium was identified (Figure 1(a)). Immunohistochemical studies identified the cyst epithelium as having pancytokeratin and progesterone receptor (PR) expressions; it was negative for carcinoembryonic antigens (CEAs), as well as negative for smooth muscle antigen (SMA) in the cyst wall indicating no smooth muscle (Figure 1(b)). The patient had no recurrence during 1 year of follow-up.

## 3. Discussion

Cutaneous ciliated cysts are asymptomatic rarely observed lesions that are usually benign. They are typically observed on the lower extremities of young girls after puberty [1, 2]. In our case, the cyst was in a previously undescribed localization on the back between the two scapulas with the age of the case, 13 years, in accordance with the literature [1]. Previous reports indicated the cysts as varying from 1 to 4 cm in size, and the cysts are generally soft in texture, fluctuating, nonhyperemic, and painless masses. In our case, the cyst was 2 × 1 cm in size, and physical examination findings were the same as those found in the literature.

Da Hess first described the lesion in 1890 on a 15-year-old girl. In 1978, Farmer and Helwig defined the cutaneous ciliated cyst in a study of the lower extremities of 11 female patients with ages ranging from 15 to 30 years [2]. The etiology is not fully known, but there are 2 theories. The first is that they are an ectopic mullerian residue since they resemble the epithelium surrounding the fallopian tubes in girls and grow with hormonal stimulation after puberty. The majority are observed as mullerian heterotrophy. According to this theory, the fallopian tubes develop from paramesonephric channel remnants in the 6-7th weeks of gestation in the embryologic period [5]. Therefore, these cells may migrate to the waist, anterior abdominal wall, lower extremities, scalp, fingers, heels, or knees during this period. These cells grow and form a cyst with the hormonal stimulation after puberty [1, 5, 6]. The more frequent incidence in girls, clear occurrence after puberty, positivity for sex hormone receptors, estrogen receptor (ER) and progesterone receptor (PR), and positivity and negativity for carcinoembryonic antigen (CEA) on immunohistochemical staining support the mullerian heterotrophy theory [6]. Additionally, they may be observed on the cheek, scrotum, and shoulders of males. The second theory is that they are ciliated metaplasia of eccrine glands. Cutaneous ciliated cysts may be observed in different areas due to vascular and lymphatic distribution [1, 3].

Some authors have considered the term “cutaneous ciliated cyst” as a cause of possible confusion and preferred to use the term “cutaneous mullerian cyst.” Some have distinguished ciliated cysts into 2 subgroups as mullerian cysts and ciliated cutaneous eccrine cysts. Cutaneous ciliated cysts with ER and PR positivity are cutaneous mullerian cysts, while negative cysts are classified as cutaneous eccrine cysts [6]. Regarding the definition and nomenclature controversies, there is a need for further studies to create a definite pathophysiologic classification; however, there are few case studies reported in the literature.

In summary, cutaneous ciliated cysts are primarily observed in young girls approaching reproductive age involving frequently lower extremities. These cysts may be confused with inclusion cysts, lipomas, adnexal cysts, bronchogenic cysts, or pilonidal cysts. Although the physiopathology of the cutaneous ciliated cysts are still not fully understood, surgical excision and immunohistochemical studies of the specimen are crucial in order to make a definitive diagnosis.
